# “If we ask, we must act”: co-designing the implementation of the EQ-5D-Y-5L as a Paediatric Patient Reported Outcome Measure in Routine hospital Outpatient Care for Kids to meaningfully impact clinical visits (P-PROM ROCK Phase 2)

**DOI:** 10.1007/s11136-025-03996-x

**Published:** 2025-05-31

**Authors:** Renee Jones, Kim Dalziel, Harriet Hiscock, Karen McLean, Diana Truong, Andrew Tuan Phuong Dao, Kathe Holmes, Kath Feely, Jessica Taranto, Abby Palmer, Tanya Wolfe, Penny Richards Fowler, Kirsten Pini, Max Pini, Einat Martonovich-Lantsberg, Joanna Lawrence, Misel Trajanovska, Adele Berry, Valerie Sung, Sebastian King, Nancy Devlin

**Affiliations:** 1 Melbourne Health Economics, University of Melbourne, Melbourne, Victoria Australia; 2 Health Services and Economics, Murdoch Children’s Research Institute, Parkville, Victoria Australia; 3 Centre for Community Child Health, The Royal Children’s Hospital, Parkville, Victoria Australia; 4 Department of Paediatrics, The University of Melbourne, Melbourne, Victoria Australia; 5 Policy & Equity, Murdoch Children’s Research Institute, Parkville, Victoria Australia; 6 Digital Innovation Team, The Royal Children’s Hospital, Parkville, Victoria Australia; 7 Department of Informatics, The Royal Women’s Hospital, Parkville, Victoria Australia; 8 Department of Gastroenterology, The Royal Children’s Hospital, Parkville, Victoria Australia; 9 Allied Health and EMR, The Royal Children’s Hospital, Parkville, Victoria Australia; 10 Department of Paediatric Surgery, The Royal Children’s Hospital, Parkville, Victoria Australia; 11 Surgical Research Group, Murdoch Children’s Research Institute, Parkville, Victoria Australia; 12 Colorectal and Pelvic Reconstruction Service, The Royal Children’s Hospital, Parkville, Victoria Australia; 13 Clinical Sciences, Murdoch Children’s Research Institute, Parkville, Victoria Australia; 14 Patient Advocate/Consumer Expert, Melbourne, Victoria Australia; 15 Centre for Digital Transformation, The University of Melbourne, Melbourne, Victoria Australia; 16 Department of Complex Care, The Royal Children’s Hospital, Parkville, Victoria Australia; 17 Prevention Innovation, Population Health, Murdoch Children’s Research Institute, Parkville, Victoria Australia

**Keywords:** Patient reported outcome measures, PROMs, Clinical care, Implementation, Co-design, Pediatrics, Paediatrics, EQ-5D-Y-5L, Consumer involvement, Quality of life

## Abstract

**Purpose:**

To co-design use of the EQ-5D-Y-5L, a generic Paediatric Patient Reported Outcome Measure (P-PROM), in Routine Outpatient Care for Kids (ROCK), maximising its impact on patient-clinician visits.

**Methods:**

This Phase 2 co-design study was guided by the co-design framework for public service design and Double Diamond model. Data collection involved facilitated workshops (building on Phase 1), followed by feedback and optimisation sessions. Participants included service providers (doctors, nurses, allied health and medical record staff), adolescents, and caregivers with lived experience of providing or receiving outpatient care at a tertiary paediatric hospital in Australia.

**Results:**

Five co-design workshops, nine feedback, and two optimisation sessions were conducted with nine service providers, two adolescents, and three caregivers. Co-design participants created resources to introduce EQ-5D-Y-5L as a ‘general health tracking questionnaire’ and explain its purpose. EQ-5D-Y-5L responses were designed to be displayed by item. A display of results over time was also designed. A patient empowerment approach was taken with regards to flagging specific EQ-5D-Y-5L items for discussion with clinicians, whereby patients or caregivers control which items are flagged. To ensure clinical review and action of EQ-5D-Y-5L responses, resources, including clinician training, clinician decision support tool, and matching patient booklet and resource pathway, were co-designed. Combined, these design elements make up the P-PROM ROCK Program.

**Conclusion:**

Consumer engagement produced important insights that would’ve otherwise been missed, ensuring the P-PROM ROCK Program empowers patients. For generic P-PROMs to meaningfully impact patient-clinician visits, supports and resources are required to ensure clinical review and action.

**Supplementary Information:**

The online version contains supplementary material available at 10.1007/s11136-025-03996-x.

## Plain English Summary

When children receive care from doctors, nurses and other healthcare providers, it can be helpful for them or their parents to tell their clinician about how their/their child’s overall health and quality of life is going. This can be done using child friendly health questionnaires, these are also known as paediatric patient reported outcome measures (P-PROMs) or health related quality of life questionnaires. However, we don’t know how we should integrate these questionnaires into care in a way that will meaningfully improve healthcare for children. The point of this study is to work together with young patients, their caregivers, and their healthcare providers to understand the best way to integrate these questionnaires into the care of children in Australia. This study found that simply asking a child or their caregiver to complete the health questionnaire is unlikely to improve healthcare for children. To meaningfully improve care, pathways and supports are needed to help families easily understand and complete the questionnaires, and for clinicians to ensure these questionnaires are looked at and responded to appropriately in appointments. This study designed a program, known as the P-PROM ROCK Program, that outlines how these questionnaires should be integrated into care alongside resources and supports.

## Introduction

Patient Reported Outcome Measures (PROMs) are validated tools used to understand a patient’s health, functioning, or quality of life from their perspective [1]. Paediatric PROMs (P-PROMs) are specifically designed for use with children and young people [2]. Where possible, P-PROMs are reported by the child themselves (child self-report) however, where the child is too young or not able to self-report, P-PROMs may be reported by the caregiver or parent (caregiver/parent proxy report) [3]. PROMs can be used in a range of contexts, including routine clinical care, population health research, clinical trials and registries, and health technology assessment [1, 4–6]. While the use of PROMs in research and health technology assessment is well established, their use in routine clinical care is more recent [4, 7]. PROMs can be a way to support patient-centred clinical care by formally bringing the patient’s perspective of their health to the clinical visit [4, 7]. When PROMs are implemented with the purpose of improving the patient-clinician visit, PROM data can improve communication, decision-making, patient engagement, and satisfaction [4, 8]. PROM data can also be used at the health system level to inform quality improvement and policy decisions [4]. Despite some uptake and evaluation of PROMs in adult clinical care, there has been limited uptake in paediatrics [9, 10].

Evidence suggests that P-PROMs with fewer items and good measurement properties could lead to improved response rates and lower patient burden [1, 11]. The EQ-5D-Y-5L is a short generic P-PROM with five items that has been shown to be easy and quick to complete [12–14]. Recent evidence comparing the performance of common generic P-PROMs in Australian children identified the EQ-5D-Y-5L as having one of the strongest psychometric performances [15]. Although the EQ-5D-Y-5L has not been trialled in routine clinical care, the adult version of the instrument, the EQ-5D-5L, has been found feasible for use in adult routine clinical care [16]. Given these potential advantages combined with the availability of funding support from the EuroQol Research Foundation, the EQ-5D-Y-5L was selected as the tool to explore in P-PROM ROCK study.

Phase 1 of the P-PROM ROCK study qualitatively explored patient, caregiver and service provider perspectives regarding the potential use of EQ-5D-Y-5L in routine clinical paediatric care [17]. Results from Phase 1 identified that although stakeholders are supportive of using a generic P-PROM such as the EQ-5D-Y-5L, simply collecting EQ-5D-Y-5L data was considered unlikely to have a meaningful impact on the individual patient visit [17]. Stakeholders highlighted that careful consideration is needed to ensure families are supported in completing EQ-5D-Y-5L prior to their clinical visit and emphasised that service providers need to be equipped to use and act on the data captured [17]. Phase 1 also identified that the use of a single score or preference weighted scoring to summarise the data, as is common to the use of such instruments in economic evaluation [18–20], would likely not be the most useful in individual clinical care: participants preferred their responses to the EQ-5D-Y-5L to be displayed by item [17]. Hence, more research is needed to understand exactly how the EQ-5D-Y-5L should be implemented, including how data from it should be scored and displayed in routine clinical care to ensure it is both feasible and useful.

The International Society for Quality of Life Research (ISOQOL) has developed a user guide for implementing PROMs in clinical practice [21]. The guide and accompanying manuscript note that a PROM program is more likely to be successful if there is meaningful and substantial engagement with stakeholders [21, 22]. This is consistent with the wider research literature that emphasises the importance of meaningfully engaging stakeholders to enable successful implementation of health initiatives following a historical paucity of such involvement, resulting in implementation failure and research waste [23–25]. A recent systematic review by Bele et al. identified seven studies that had investigated the impact of implementing a P-PROM in routine paediatric clinical care [10]. Of these seven studies [10], only three reported engaging clinician stakeholders in the design of the P-PROM intervention or implementation [26–28]. No studies reported engaging parents or children. Since that systematic review, only one study could be identified that reported the inclusion of children and caregivers in the co-design of a P-PROM program [29]. More research is needed that includes the perspectives of parent and child consumers, as well as service providers, in the design process. Such learnings are likely valuable to others seeking to implement P-PROMs in clinical contexts.

Building on Phase 1 [17], this paper reports Phase 2 of the P-PROM ROCK study. This study aims to co-design use of the EQ-5D-Y-5L, a generic P-PROM, in Routine Outpatient Care for Kids (P-PROM ROCK), to maximise impact on patient-clinician visits. A further aim was to reflect and report on the co-design process. The P-PROM ROCK Program developed from these efforts will be piloted and evaluated in Phase 3.

## Methods

### Study design

**Fig. 1:**
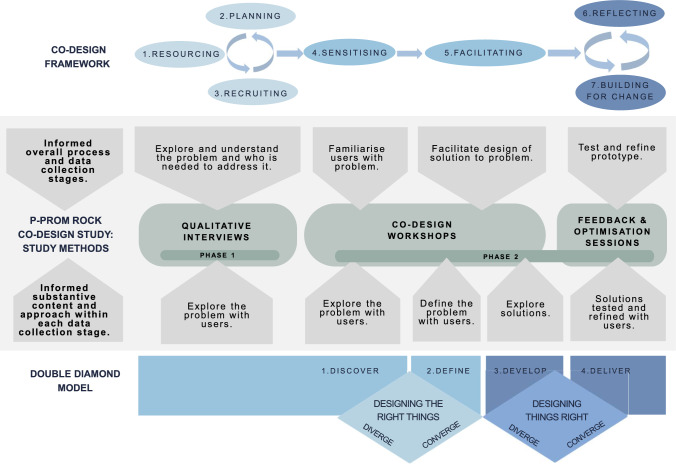
Application of methodological frameworks to P-PROM ROCK study methods. *Note* Graphic was created using Canva (canva.com). Figure is adapted from the co-design framework for public service design and the Double Diamond Model to describe how both frameworks influenced study methodology [31, 32]

This study utilised co-design methodology. Co-design is an iterative process involving collaboration between key stakeholders [30]. In this study, two complementary methodological frameworks were utilised (Fig. 1): (1) a co-design framework for public service design [31], and (2) the Double Diamond model [32]. The co-design framework for public service design includes seven steps: resourcing, planning, recruiting, sensitising, facilitating, reflecting, and building for change [31]. This framework informed the studies overall process and data collection, including co-design workshops, feedback sessions, and optimisation sessions. The Double Diamond model includes four phases: discover, define, develop, deliver [32]. Although the co-design framework provided a step-by-step process, the study team felt the Double Diamond framework was needed to inform the approach taken within steps. For example, the co-design framework prompted the study team to conduct workshops, while the Double Diamond model shaped the focus of discussions within workshops. Figure 1 outlines how these two methodologies informed study methods. Further explanation is also available in the ‘Data collection‘ section.

### Setting and participants

The Royal Children’s Hospital (RCH) is the largest tertiary paediatric hospital in Melbourne, Australia. Outpatient appointments are primarily conducted in person at the hospital, although some are conducted via telehealth. Appointment times range between approximately 15–30 min. The RCH uses the Epic Electronic Medical Record (EMR) for all clinical documentation and storage of patient medical information (www.epic.com/). Patients cared for by these outpatient clinics were considered suitable for a P-PROM program given the benefit of PROMs in chronic care management [8].

Eligible participants included service providers (allied health staff, doctors, EMR analysts, nurses, and virtual care consultants), adolescents aged 12–18 years, or caregivers of children aged 2–18 years, who had experience providing or receiving outpatient care at RCH. Only one adolescent or caregiver per family were eligible.

Participants were recruited between July and August 2023. A range of recruitment approaches were utilised. Firstly, participants who took part in the qualitative interviews in Phase 1, were emailed and asked if they would like to take part. Secondly, eligible caregiver participants from the Australian Paediatric Multi-Instrument Comparison Study (P-MIC) study [33], were emailed and invited to take part in this study. To enable recruitment of adolescents aged 12 to 18 years, caregivers were asked if they were willing to have their adolescent child take part. Finally, the study team’s professional network of service providers was emailed to ask if they would like to take part. Snowball sampling was also utilised [34], whereby service providers were asked to pass on the email to colleagues in their network.

### EQ-5D-Y-5L

The EQ-5D-Y-5L is a new generic P-PROM, officially launched in September 2024 [13]. Approval was obtained to use the EQ-5D-Y-5L for this study. The EQ-5-Y-5L improves on the EQ-5D-Y-3L, increasing the number of response levels from three to five [12]. The EQ-5D-Y-5L has five items: mobility, self-care, usual activities, pain/discomfort and sad/worried/unhappy. Each item has five outcome levels on a severity scale of no problems to extreme problems or cannot do. The EQ-5D-Y-5L also includes a visual analogue scale (EQ VAS) which asks about the child’s health today on a scale of 0 (worst health) to 100 (best health) [12].

### Data collection

Data collection included co-design workshops (conducted August-November 2023), feedback sessions (conducted November 2023), and optimisation sessions (conducted November 2023). Ethics approval was obtained from The Royal Children’s Hospital Human Ethics Committee (HREC/92769/RCHM-2023).

#### Co-design workshops

The following were identified in Phase 1 as key topics to be addressed in co-design workshops: (1) scoring and displaying EQ-5D-Y-5L, (2) integrating EQ-5D-Y-5L into patient journeys and clinic workflows, (3) how to respond when a child reports a problem on EQ-5D-Y-5L, and (4) how to get patients & caregivers engaged.

Prior to co-design workshops, participants undertook a series of tasks to familiarise themselves with workshop topics, to encourage thinking and reflection on the problem (known as ‘sensitisation’ in co-design framework) [31]. These tasks included an introductory video that outlined what a P-PROM is, how and why P-PROMs are used in clinical care, what EQ-5D-Y-5L is, why we are doing the study, and what to expect from the workshops. Additionally, participants were asked to complete the EQ-5D-Y-5L. Additional sensitisation tasks were conducted within workshops to introduce participants to topics, further detail on this is available in the Results.

To meet the requirements of participants, workshops were held online, in person and/or via a hybrid format. Zoom, including ‘break out rooms’ and ‘Zoom Whiteboard’, was used for online and hybrid workshops (Zoom Video Communications Inc., 2016, www.zoom.us/). The interactive presentation software Mentimeter was also used to obtain general anonymous thoughts from the group (Mentimeter North America Inc., www.mentimeter.com). All workshops were recorded, and notes were taken.

Workshop facilitation was primarily led by RJ, however, other members of the research team, including HH, KD and ND, facilitated some sections of the workshops. During each workshop, participants were introduced to workshop topics and given an opportunity to discuss as a group. This initial discussion was not constrained by feasibility (known as ‘discover’ in the Double Diamond Model), participants could contribute verbally, via the chat function, or anonymously via Mentimeter [32]. Following this, facilitators prompted participants to consider feasibility and consensus (known as ‘define’ in Double Diamond Model), similar to the initial discussion, different communication options were provided to participants [32]. After initial discussions, participants were given an activity. These activities were often creative [35, 36]. For example, in the first workshop, participants were given a case vignette of a child with a condition and a specific EQ-5D-Y-5L response, they were then given a blank section of Zoom Whiteboard to draw how they thought this patient’s EQ-5D-Y-5L response should be displayed in a clinical context (See Supplementary Fig. 1a). Where possible, participants were given the opportunity to complete the activity alone, enabling independent thought. Everyone then discussed their activity output in the group (known as ‘develop’ in Double Diamond Model) [32]. Following this, participants were prompted to engage in consensus discussions, moving towards a final prototype (known as ‘deliver’ in Double Diamond Model) [32]. Additionally, to minimise potential power imbalances between participants during workshops, strategies from Ní Shé É et al. (2021) were adapted and utilised, [37] including (1) the use of creative based activities, (2) lay and accessible language, (3) ensuring all participants were compensated equally for their time, (4) providing opportunities and appropriate supports to ensure equal participation (e.g., anonymous ways to share thoughts or ways to share thoughts in non-group settings), and (5) ensuring all participants were provided with the opportunity to participate or be acknowledged in any publications, presentations and achievements that result from this work.

#### Feedback and optimisation sessions

Data from the co-design workshops was used by the study team to develop draft prototypes. Several rounds of design iteration and feedback proceeded to reach a prototype that would best fit the local context. Feedback was obtained from participants on these prototypes at the next workshop (known as ‘reflecting’ and ‘building for change’ steps in co-design framework) [31]. Adolescents and caregivers were also offered the opportunity to provide further feedback via online interviews and service providers via email. Following the co-design workshops and feedback sessions, a full prototype was drafted.

Optimisation sessions were conducted to test the prototype in mock patient-clinician visits, assessing performance in a real-world context, and making final refinements (known as ‘reflecting’ and ‘building for change’ steps in co-design framework) [31]. During these mock patient-clinician visits, a mock patient and mock clinician were asked to follow a case vignette and act out a mock outpatient visit that included the P-PROM ROCK Program. Mock patients and clinicians were asked to reflect out loud on the process of the P-PROM ROCK Program as they were going through it, and key points from this were noted down by a member of the research team. At the end of the session, key points were discussed and clarified between mock patients, clinicians, EMR specialists, and the research team to establish a list of changes to be made to the prototype.

## Results

Five co-design workshops, nine feedback sessions and two optimisation sessions were conducted.

### Participants

Across the five co-design workshops, two adolescent patients, three caregivers of patients, and 11 service providers participated. The two adolescent patients were aged 14 and 16 years old. One adolescent took part alongside their parent, and the other did not. The three caregivers were mothers of patients aged between seven and 11 years. Adolescent and caregiver participants have experience receiving care from endocrinology, continence, behaviour, development, asthma, sleep, and neurology outpatient clinics. The 11 service providers included four doctors, three nurses, and four EMR specialists, of which two also have allied health experience. These service providers have experience providing care to children in asthma, sleep, continence, colorectal surgery, gastroenterology transplant, and hearing outpatient clinics, as well as providing specialist EMR support across RCH. Not all participants attended all workshops (see Table 1).

**Table 1:** Summary of workshop participants, format, topics, sensitisation tasks, activities, and outputs

**Workshop**	**Participants**	**Topic(s)**	**Sensitisation**	**Activities**
**Workshop 1, 2-hours, online.**	Adolescent (n = 1), Caregivers (n = 3), & Service Providers (n = 6). Facilitators (n = 4).	· Scoring & displaying EQ-5D-Y-5L. · Integrating EQ-5D-Y-5L into patient-/ clinic work- flows.	· Presentation on scoring and display options & examples.(16, 38, 39) · Group asked to vote on preferred options & to rank EQ-5D-Y-5L items.	· Design ideal EQ-5D-Y-5L display using Zoom Whiteboard (Supplementary Figure 1a and b). · Design patient journey and clinic workflow via group discussion with use of case vignette.
**Workshop 2, 2-hours, online.**	Adolescent (n = 2), Caregivers (n = 3), & Service Providers (n = 9). Facilitators (n = 4).	· Scoring & displaying EQ-5D-Y-5L (Feedback) · How to respond when a child reports a problem on EQ-5D-Y-5L. · How to get patients & caregivers engaged.	· Introduced workshop 1 draft prototype. · Presentation on ways to respond if a child reports a problem. · Vote on preferred ways to respond & anonymous open text to group. · Presentation on examples of how patients previously engaged in PROMs.	· Refine prototype from workshop 1 - via group discussion. · Design ideal plan for introducing patients and caregivers to completing and using EQ-5D-Y-5L – via group discussion. · Design where in EMR system the display should appear – via live demonstration in EMR system.
**Workshop 3, 1-hour, in person.**	Service Providers only (n = 4). Facilitators (n = 2).	· How to respond when a child reports a problem on EQ-5D-Y-5L (focus on resources).	· Presentation of examples of clinician P-PROM resources from Netherlands.(39) · Introduced workshop 2 draft prototype.	· Comment likes & dislikes in margins of a printed draft prototype. · Design ideal resource via group discussion & using pen/paper.
**Workshop 4, 1-hour, in person.**	Service Providers only (n = 4). Facilitators (n = 2).	· How to respond when a child reports a problem on EQ-5D-Y-5L (focus on clinician training).	· Presentation of PROM clinician training literature.(40) · Introduction to example training program from Netherlands.(39, 40)	· Group reflection on their likes & dislikes of example training. · Design training challenges, goals, length, format, & content.
**Workshop 5, 1-hour, hybrid (in person and online).**	Service Providers only (n = 5). Facilitators (n = 2).	· Integrating EQ-5D-Y-5L into patient-/ work- flows (Feedback). · How to respond when a child reports a problem on EQ-5D-Y-5L (Feedback).	· Presentation of journey map draft prototype. · Presentation of resources draft prototype. · Presentation of plan for trial design.	· Discuss ways journey map (patient and clinical workflow) could be improved. · Discuss ways resources (clinician only and patient/caregiver) could be improved.

### Co-design workshops

Two 2-h online workshops were conducted with service providers, adolescents, and caregivers. An example of a creative workshop activity output is available in Supplementary Fig. 1b. It was then identified that additional workshops were required to further design aspects of the solution that related to service providers only (e.g., clinician specific training and clinician specific resources). Hence, an additional three 1-h in person/hybrid workshops were conducted with only service providers. The decision to add additional workshops for service providers only was discussed with all participants in the second workshop and all agreed this was an appropriate course of action. Workshops format (online/in person/hybrid) was decided based on the ability of participants to attend a workshop, if participants could only attend online then the workshop was online, whereas if most participants could attend in person as was the case with the clinician only workshops, these were hybrid or in person. A summary of these five workshops is described in Table 1.

### Feedback and optimisation sessions

Five feedback sessions were conducted within workshops. Four feedback sessions were conducted after workshops via individual online meetings with participants (n = 3 caregivers and n = 1 adolescent). Additional feedback was also obtained from service providers via email. Finally, two optimisation sessions were conducted where a mock patient-clinician visit was acted out by service providers (n = 2, one was from the co-design group and the other was not) and mock patients (n = 2, not from the co-design group). Two case vignettes were followed. These mock sessions required patients to use the EMR Patient Portal to complete the EQ-5D-Y-5L, and clinicians to refer to answers via the EMR (EPIC) in a mock consultation.

### Designing the different prototype elements

Participants designed different prototype elements, guided by the co-design workshop topics. A description of the process to design these different elements is described by topic in Table 2.. Further design aspects that were not feasible for inclusion in the final prototype are described in Supplementary Table 1.

**Table 2:** Description of P-PROM prototype element development, by workshop topic

**Topic**	**Description of prototype design element **	**Reasoning for design / response from participants**
**Scoring & displaying EQ-5D-Y-5L**	EQ-5D-Y-5L single time point table display: · Two columns, one with the EQ-5D-Y-5L items (wording) & the other with the response level selected (wording). · Date of completion and who completed it (i.e., child or caregiver). · Visible to patient or caregiver immediately after completion in patient portal or on via paper handed to them. · Visible to service providers in EMR system.	· Table format easy to understand & interpret. · Displaying responses by item was preferred as this easily highlights areas of concern. · Summarising responses into a single score decreased interpretability. · Important to know who completed, as may impact clinical conversation. · Important to know when completed, due to short recall period & change.
	EQ-5D-Y-5L line graph display over time: · Each EQ-5D-Y-5L item appears as a separate line. · Higher score reflects better health, i.e., no problems = five & extreme problems/unable to = one. · Y axis numbered 1-5 & X axis is date of completion. · Visible to service providers by clicking into a section of EMR system that provides summaries of patient information.	· Line graph perceived as concise summary of responses over time. · Higher point on a graph instinctively meant ‘better’ or ‘good’, whereas a lower score instinctively meant ‘worse’ or ‘bad’. Hence the usual level sum score approach was flipped. · Preference for labelling the Y axis with the response levels was not possible in the available infrastructure. Hence, the simple labelling approach was used. · Table format display considered easier to interpret, hence was the first summary provided to patients, caregivers and service providers.
	Flagging system: · Patient or caregiver to select which EQ-5D-Y-5L item(s) they would like to discuss with their service provider in appointment. · Item(s) they identify to appear in the clinician table display in red bold text with yellow highlight to draw the clinician’s attention to this.	· Designed to give autonomy and power to patient. Automated approaches to flagging felt to cause external negative value judgement & may highlight a non-concern. · For patients & caregivers, red alerts were associated with ‘bad’. Negative framing reminded participants of a ‘school report’ or something they might be ‘failing’. Hence, alert does not appear in patient/caregiver display. · The visual alert in the clinician display was appropriate as similar visual alerts are used for other abnormal results that require action.
**Integrating EQ-5D-Y-5L into patient- work- flows**	Journey map (patient journey and clinical workflow): · Completion of EQ-5D-Y-5L attached to outpatient visit. Service provider responsible for visit is responsible for reviewing EQ-5D-Y-5L response. · Patients/caregivers able to complete up to 7 days before via patient portal system (online, links to EMR) or paper. · Designed table display to appear in the main outpatient clinic view in the clinician EMR. Designed line graph to appear in the summary view in the clinician EMR.	· Important a service provider was responsible for reviewing response and this was only available at clinic visits (i.e., monitoring responses between visits was not feasible). · Completing prior to entering appointment room is important as no time available during the appointment. Also means families can complete in more relaxed environment. · Although portal completion was preferred, it was important to provide options. · Table display designed to appear in EMR outpatient view as this is where service providers already go to review results and take notes for that visit. · Although service providers less familiar with the summary view on EMR, they felt with appropriate training and resources they would be able to locate this.
**How to respond when a child reports a problem on EQ-5D-Y-5L**	Clinical decision support tool (1 page document for clinicians): · Four sections: 1) locate (where to locate EQ-5D-Y-5L response), 2) identify (how to identify a problem), 3) discuss (how to engage in a conversation about response), and 4) act (how to act or respond). · ‘Act’ section has three options: 1) condition related support (green box), 2) urgent support (red box) and 3) mild or moderate concern appropriate for community support (blue box).	· Service providers not sure how to engage in quick conversation about EQ-5D-Y-5L responses, hence discussion prompts included to help navigate conversation swiftly. · Service provider scope in outpatient clinics is specific & might not be best placed to support children with general concerns. Hence, pathway to community supports designed.
	Resource pathway documents (for clinicians and patients/caregivers): · Resource pathways for urgent concerns (clinician only – red document), mild or moderate concerns (patient/ caregiver – blue document) · All resources cover ways in which families can get support for the different EQ-5D-Y-5L items. · Colour-coded to match with the clinician decision support tool. · Patient/ caregiver to automatically receive mild/moderate resource pathway document directly after EQ-5D-Y-5L completion. · Paper copies to have QR codes, online copies to have links.	· Participants encouraged the red and blue resources to be designed in collaboration with the experts from RCH. Consequently, the mental health team, allied health team, and pain team were all consulted in the design of these resources. · For EQ-5D-Y-5L to be meaningfully used in clinical visits, it was important there were clear strategies to support children or caregivers where the child had a problem on one of the EQ-5D-Y-5L item(s) and wanted support for this. · Some patient and caregiver participants felt that general resources were not always helpful to them. Hence, it was made clear to service providers in the training & clinical decision support tool that responses should be discussed.
	Clinician training (30-60 minutes): · Conducted during an existing meeting time slot. · Content includes: 1) introduction to P-PROM ROCK Study, 2) introduction to PROMs, 3) why PROMs in clinical care 4) introduction to the EQ-5D-Y-5L, 5) where to locate in EMR, 6) what to do if a child has a concern, & 7) case examples.	· Service providers needed training to use the EQ-5D-Y-5L in clinical care in addition to an introduction to the different resources available. · Training designed to overcome potential obstacles (time poor, funded time), and to address key goals (promoting P-PROMs, practical aspects, and what to do if a child has a problem). · Participants were clear that most of the training should be dedicated to point 5 (where to locate responses) and 6 (how to act on responses).
**How to get patients & caregivers engaged**	Information package for patients/caregivers: · Sent to families before appointment. Short summary available directly before & after EQ-5D-Y-5L completion. · Includes: 1) why they are being asked to complete the EQ-5D-Y-5L, 2) how this might benefit them/their child, 3) how they should complete it, 4) how long it will take, 5) what questions are included, 6) who will be able to see their responses, & 7) what will be done with their responses. · Available in both written & video (with cartoons) format as participant information and consent form (PICF).	· Felt all information should be provided in advance so families could make an informed choice. · Felt that key information from this package should be included directly before and after completing the EQ-5D-Y-5L as a reminder. · Information package should be easy for families to understand and engage with, hence both a written and video version of the information was designed. · Given the context of the co-design workshops was to inform a pilot trial, participants used this introduction package design to inform a trial participant information and consent form (PICF) and video.
	EQ-5D-Y-5L introduced as ‘general health tracking questionnaire’.	· The terms ‘EQ-5D-Y-5L’ or a ‘quality of life questionnaire’ were associated with something very serious by patients/caregivers that could mean possible judgment. Hence, new language was designed.

### Final co-designed prototype (P-PROM ROCK Program)

**Fig. 2:**
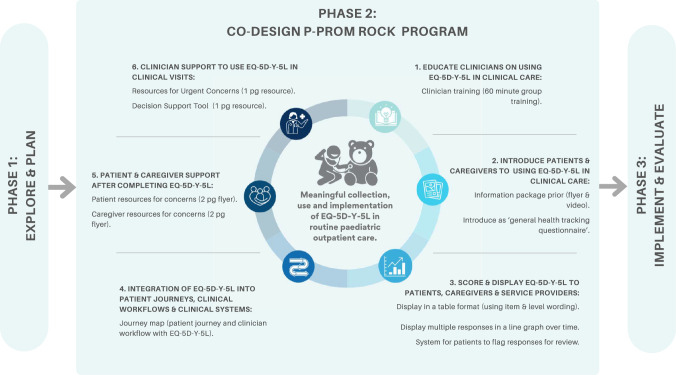
Final P-PROM ROCK Co-designed Prototype (P-PROM ROCK Program). *Note* Graphic was created using Canva (canva.com)

The different design elements were combined to make a final prototype, known as the P-PROM ROCK Program. The P-PROM ROCK Program includes six key elements which are summarised in Fig. 2 and described below. Participants also contributed to the planned evaluation of the P-PROM ROCK Program, deciding on key outcomes and aspects of trial design, which will be explored in Phase 3.

1. **Educate clinicians on using EQ-5D-Y-5L in clinical care—Clinician training**
  Education of clinicians on using EQ-5D-Y-5L via a 60 min, in person, small group training session. The training sessions are to be conducted in an existing meeting or existing blocked out clinical time where possible.
2. **Introduce patients and caregivers to using EQ-5D-Y-5L in clinical care—Information package**
  Patients and caregivers are introduced to using EQ-5D-Y-5L prior to their appointment via an information package available in flyer and video format. Additionally, core information is provided directly prior to and after completing the EQ-5D-Y-5L (Supplementary Fig. 2). The EQ-5D-Y-5L is introduced in all documentation as the ‘general health tracking questionnaire’.
3. **Score and display EQ-5D-Y-5L to patients, caregivers and service providers—EQ-5D-Y-5L displays**
  EQ-5D-Y-5L responses are displayed to patients/caregivers, and clinicians in two different displays in the EMR that indicates who completed the information and on what date: A table format using item and level wording (Supplementary Fig. 3), and as multiple responses in a line graph over time, where a higher score reflects better health (Supplementary Fig. 4). EQ-5D-Y-5L items scores usually run in the other direction, where a higher score reflects worse health, so for this longitudinal display, scoring was flipped. Additionally, to empower the patient, they or caregiver can indicate which EQ-5D-Y-5L items they would like to discuss with their service provider (Supplementary Fig. 5), which appears as an alert (red text with yellow highlight) in the service provider display (Supplementary Fig. 3).
4. **Integration of EQ-5D-Y-5L into patient journeys, clinical workflow and clinical systems—Journey map**
  Completion of EQ-5D-Y-5L is attached to a specific outpatient visit and the clinician allocated to that visit is responsible for reviewing the response. Patients/caregivers can complete the EQ-5D-Y-5L up to seven days before their outpatient appointment visit, and they receive a reminders (if required) at seven days, two days, and in visit waiting room to complete the EQ-5D-Y-5L. Patients/caregivers complete EQ-5D-Y-5L via the patient portal integrated into the EMR system (Epic MyChart) or on paper on the day of the appointment. Supplementary Fig. 6 provides a visual overview.
5. **Supports for patients and caregivers after completing EQ-5D-Y-5L—Family resources**
  Directly after completing EQ-5D-Y-5L, patients/caregivers automatically receive a list of resources via the online system used to complete it (Epic MyChart) or via paper if they completed a paper version. The resources explain how they could access support if they had a concern with one of the EQ-5D-Y-5L items. There are two versions of this to better meet the needs of the recipient. If the child self-reports, they receive a young person resource, and if the caregiver proxy reports they receive the caregiver resource (Supplementary Fig. 7).
6. **Supports for clinicians to use EQ-5D-Y-5L in clinical visits—Clinician resources**
  Clinicians receive a Clinical Decision Support Tool that includes where to locate the result, how to identify a problem, how to engage in a conversation about the results, and suggestions on how to action or respond to a concern (Supplementary Fig. 8). Clinicians additionally receive an urgent support resource that outlines available service options if a child has an urgent concern with one of the EQ-5D-Y-5L items (Supplementary Fig. 9). The final section of the clinical decision support tool cross references to the corresponding sections of the family and urgent clinician resources.

## Discussion

In this study, adolescent patients, caregivers of patients, and service providers co-designed use of the EQ-5D-Y-5L in routine paediatric outpatient care, developing the P-PROM ROCK Program. This co-designed P-PROM ROCK Program acknowledges that simply collecting a generic P-PROM in a clinical context, without additional resources to support its implementation, completion, or use, is unlikely to have a meaningful impact on patient-clinician visits. Consequently, the P-PROM ROCK Program is multi-dimensional, wrapping around the patient, their caregiver, and service providers, delivering support at different points along the journey of EQ-5D-Y-5L implementation, completion and use in routine clinical care. The P-PROM ROCK Program includes clinician and patient education, a clinically meaningful display of EQ-5D-Y-5L responses, a patient-centred approach to flagging a concerning response, integration into local workflows and systems, and resources to support patients, caregivers, and service providers to respond to EQ-5D-Y-5L information. Engaging patients, caregivers of patients, and service providers in the co-design process was feasible, with all participants able to meaningfully contribute to the design. Furthermore, this engagement was considered essential to generating a user-centric design.

This is the first published study to report on the co-design process of a P-PROM program that includes patients, their caregivers, and service providers. Previous studies had either not involved any end users in their P-PROM program design, only included service providers [26–28], or had not reported the details of the co-design process [29]. This is also the first study to report use of EQ-5D-Y-5L in clinical settings [10]. The inclusion of adolescents and their caregivers in this co-design process is a strength of this study and was pivotal to ensuring the design was acceptable and applicable to patients and their families. It was important to include both adolescents and caregivers, as their voices differed. Adolescent participants brought joy and creativity to the co-design team. A service provider participant reflected that the adolescents’ creativity served as a reminder they were designing something for a children’s hospital. Bringing providers together with patients and caregivers kept a focus on patient relevance and guided decisions back toward patient benefit. After the workshops, several participants reflected on the benefits of this joint involvement, noting this broadened their thinking beyond their individual experience. Involvement of service providers in the co-design was critical to gain their buy-in. Many co-design processes keep service providers and patients separate, whereas our experience suggested significant benefits of bringing these groups together. Another strength of this study was the use of two methodological frameworks, [31, 32]. These two frameworks were complementary and without both it is likely important steps and approaches would have been missed. For example, without the seven-step co-design framework, key steps in the co-design process may have been missed, and without the Double Diamond Model, certain discussions or ideas may have been missed [31, 32]. Additionally, as part of Phase 1 of this study, the recruitment of participants for this co-design study was given careful consideration as part of the ‘recruitment’ step of seven-step co-design framework. As a result, participants in this co-design study reflected a range of different medical areas (chronic, episodic, acute, surgical) and roles (service providers, patients, caregivers). Moreover, within these groups, careful consideration was given to ensure representation from different key sub-populations. For example, some patients have more complexity and hence have contact with a wide range of different health services, whilst others only have contact with a single service, and participants with these different experiences were included in the study.

Including patients, caregivers, and service providers together in workshops was not without its challenges. Firstly, the sample size had to be kept to a size that would enable all participants to share their thoughts in a whole group context. Secondly, scheduling a time for workshops that all participant groups could attend was difficult. Consequently, only two online co-design workshops were able to be conducted with all participant groups present and feedback was largely obtained on an individual basis. This may have resulted in different feedback than a group context. A limitation to hosting the workshops online was that quite a few participants were not familiar with the technology which may have impacted their ability to fully engage, especially with creative tasks. Future studies may consider meeting with participants one-on-one beforehand to ensure they are confident with the technology. Potential power imbalance between participants is another limitation of this study; however, efforts were made to minimise this, including ground rules, having designated times where services providers and patients/caregivers were separated, and giving participants different ways to engage in discussion, including anonymously. One adolescent participant reflected that the creative activities were a great way to reduce power imbalances. Additionally, one caregiver participant reflected on how respectful all participants were, even when disagreeing on something. Several participants reflected on factors that contributed to this respectful environment where they felt comfortable sharing, including: (1) consistent relationship with facilitator throughout, (2) same participants in each workshop, and (3) trust in facilitator. Service providers who took part in this study may also reflect those who are early adopters and more open to the use of P-PROMs in clinical care, hence, future research should explore if these findings hold true with service providers who are more hesitant about the use of P-PROMs. Non-English-speaking participants were not represented in this study, which is a limitation and should be a focus of future research. Additionally, it is unknown how elements of this design may be generalised to other age groups, clinical settings, and geographic contexts. This should also be the focus of future research.

When the P-PROM ROCK Program is compared with other existing PROM programs, there are important differences and similarities. In terms of displaying PROMs for use in routine clinical care, different programs have taken different approaches. For example, an adult kidney care PROM program in Canada, known as the EMPATHY trial, displays EQ-5D-5L responses in a report card style, with different response levels categorised as a tick, a caution sign or a stop sign [16]. This differs to the P-PROM ROCK Program display, as participants wanted to avoid displays that looked like school reports or framing responses in a way that could be perceived as failure (colour red in a traffic light system). They also wished to avoid applying external thresholds or value judgements to responses, preferring a system that empowers patients to decide what is flagged for discussion. A P-PROM program in the Netherlands, known as KLIK, displays PedsQL domain scores using a traffic light colour system for clinicians but not for families or children, due to similar concerns identified in this study regarding negatively framed displays [39]. When flagging results for clinicians, the KLIK system uses external thresholds to determine what is flagged as red or orange to clinicians. As discussed above, participants in this co-design study wanted patients to have control over what is flagged as an issue based on what is a concern to them, hence they designed a flagging system where the patient or caregiver is in control of what is flagged for discussion. Both programs displayed PROMs by item or domain, similar to the P-PROM ROCK Program, which may suggest this approach could be applied more widely in routine clinical care. This differs to how the EQ-5D-Y-5L is typically scored when used in clinical trials, where the aim is to facilitate use in economic evaluation, and is an important finding of this study [18]. In terms of resources and supports, service provider participants in the P-PROM ROCK study appreciated the simplicity of the decision support tool used in the KLIK P-PROM program [39]. Consequently, a very similar approach was taken in this study. However, additional supports were also generated as part of the P-PROM ROCK Program that focus on how to action concerns identified on the EQ-5D-Y-5L. These supports were categorised by level of concern: urgent (supports for clinician to action in appointment) and mild/moderate (community supports for families to action after appointment). The differences and similarities between the P-PROM ROCK Program and other programs may be due to the local context or the addition of patients in the design process.

## Conclusion

This Phase 2 study builds on our previously published qualitative Phase 1 [17], and provides a co-designed program for implementing, collecting and using a generic P-PROM, the EQ-5D-Y-5L, in clinical outpatient care (hospital-based visit), in an Australian tertiary paediatric hospital. A key finding from this study, based on the perspectives of the service providers, caregivers and adolescent patients who took part, is that if patients are asked to complete P-PROMs, there is a duty of care for this information to be reviewed and acted upon appropriately by service providers. Involving children, caregivers and healthcare service providers in the co-design of P-PROM collection processes, displays, training, and supports to respond, provides essential insights and gives P-PROMs the best possible chance of successful implementation and impact on patient care. The ultimate test of whether routine use of P-PROMs is impactful is whether the data are actively used in healthcare decisions, and whether healthcare is delivered in a way that better meets patients’ needs. The planned evaluation of the P-PROM ROCK Program, Phase 3, will provide evidence on these outcomes.

## Supplementary Information

Below is the link to the electronic supplementary material.

Supplementary file1 (DOCX 1294 KB)

## Data Availability

Data is available upon reasonable request.
