# Supplementary material for: “If we ask, we must act”: co-designing the implementation of the EQ-5D-Y-5L as a Paediatric Patient Reported Outcome Measure in Routine hospital Outpatient Care for Kids to meaningfully impact clinical visits (P-PROM ROCK Phase 2)
Source: Qual Life Res. 2025 May 31;34(8):2205–18. doi: 10.1007/s11136-025-03996-x (PMC12274246; doi:10.1007/s11136-025-03996-x)
Supplement: Supplementary file 1 — Supplementary file1 (DOCX 1294 KB) [file 11136_2025_3996_MOESM1_ESM.docx]

**Supplementary materials**

Supplementary Materials for Manuscript: “If we ask, we must act”: Co-designing the implementation of the EQ-5D-Y-5L as a Paediatric Patient Reported Outcome Measure in Routine hospital Outpatient Care for Kids to meaningfully impact clinical visits (P-PROM ROCK Phase 2).

**Contents**

[Supplementary Table 1. Description of design elements not feasible in local context, by workshop topic. 2](#_Toc194589837)

[Supplementary Figure 1a.Example of creative activity set up in workshop. 3](#_Toc194589838)

[Supplementary Figure 1b. Example output from creative activity in workshop. 3](#_Toc194589839)

[Supplementary Figure 2. EQ-5D-Y-5L Introduction. 4](#_Toc194589840)

[Supplementary Figure 3. Example of clinician display from a completed EQ-5D-Y-5L questionnaire (single time point table). 5](#_Toc194589841)

[Supplementary Figure 4. Example of clinician display from multiple completed EQ-5D-Y-5L questionnaires (over time, line graph). 6](#_Toc194589842)

[Supplementary Figure 5. Additional question used for children/caregivers to flag EQ-5D-Y-5L items to service providers. 7](#_Toc194589843)

[Supplementary Figure 6. Journey Map. 8](#_Toc194589844)

[Supplementary Figure 7. Example of Caregiver Resources. 9](#_Toc194589845)

[Supplementary Figure 8. Clinician Decision Support Tool. 11](#_Toc194589846)

[Supplementary Figure 9. Clinician Resources for Urgent Concerns. 12](#_Toc194589847)

# Supplementary Table 1. Description of design elements not feasible in local context, by workshop topic.

| **Topic** | **Description of design elements not feasible in local context** |
| --- | --- |
| **Scoring and displaying**  **EQ-5D-Y-5L.** | **Extra context:**  Participants noted that it would be ideal if patients or their caregivers could have the option to add extra context to their EQ-5D-Y-5L response by having an open text box available at the end of the EQ-5D-Y-5L. Participants felt this would save patients and caregivers needing to remember such information when the EQ-5D-Y-5L responses are discussed in the appointment and it would also potentially save clinic time by enabling a more targeted conversation in the clinical visit. Unfortunately, the ability to add extra context was not feasible in the available IT infrastructure. |
|  | **Making displays fun:**  Participants also explored ways of making the completion and display of EQ-5D-Y-5L more fun and engaging for children. Some ideas included children being able to select a cartoon that would guide them through the process of completing the EQ-5D-Y-5L and would also appear in the display or results. Unfortunately, this was also not possible in available IT platforms. Figure 2b, is a Zoom Whiteboard drawing from an adolescent participant that depicts the idea of using shapes to flag certain results as well as using cartoons or pictures to better engage children. |
|  | **Storing EQ-5D-Y-5L responses in dashboard:**  Participants, particularly patients and caregivers of patients felt that it was important for EQ-5D-Y-5L responses to be displayed alongside any other clinically relevant information, such as condition-specific P-PROMs and medication or treatment changes. Although such a dashboard was not feasible in currently available IT systems, an approach was designed whereby EQ-5D-Y-5L responses would be stored within systems where this other clinically important information was available. |
| **Integrating the EQ-5D-Y-5L into patient- / work- flows.** | **Frequency of EQ-5D-Y-5L completion, between visits**:  Adolescent patient and caregiver participants discussed the benefits of completing the EQ-5D-Y-5L just before each visit as well as at multiple time points between each visit. They noted that as they often have outpatient appointments every three to six months, being able to monitor and pick up on any issues between visits would be helpful. However, it was decided that current models of care were not suitable to support patients completing the EQ-5D-Y-5L between visits, as there was no clinical resourcing to review and action any patient EQ-5D-Y-5L responses between visits. Service provider participants felt strongly that there was a responsibility to review and action EQ-5D-Y-5L responses once they are submitted by the patient. |
| **Engaging families in**  **EQ-5D-Y-5L.** | **Other languages:** Participants explored having the information package available in different languages. Although this would have been ideal, it was not feasible for this project. |

# Supplementary Figure 1a. Example of creative activity set up in workshop.


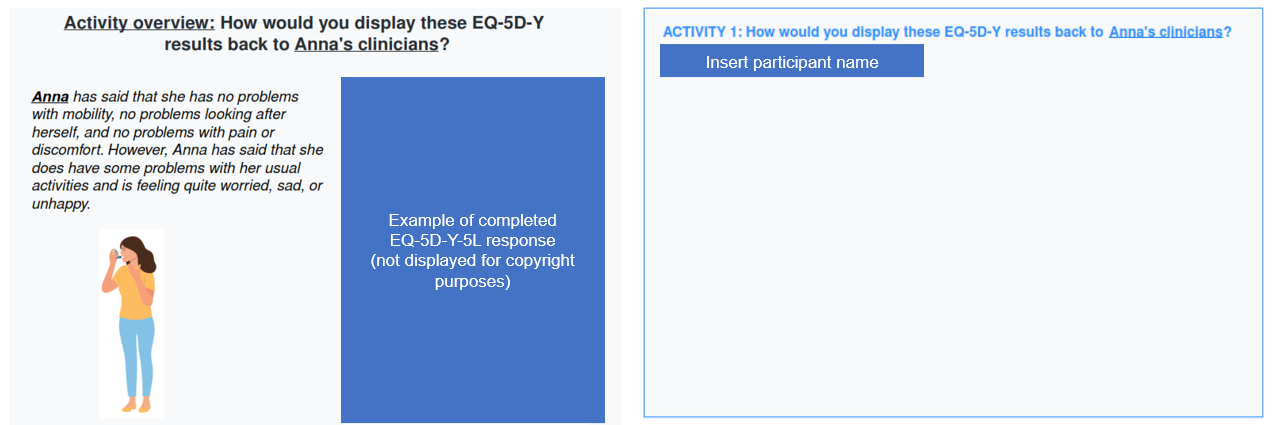


***Note:*** *This figure shows how one of the workshop activities was set up, this activity provided participants with a case vignette and asked them to draw how they would display the EQ-5D-Y-5L responses back to clinicians. This activity was conducted in Zoom Whiteboard.*

# Supplementary Figure 1b. Example output from creative activity in workshop.


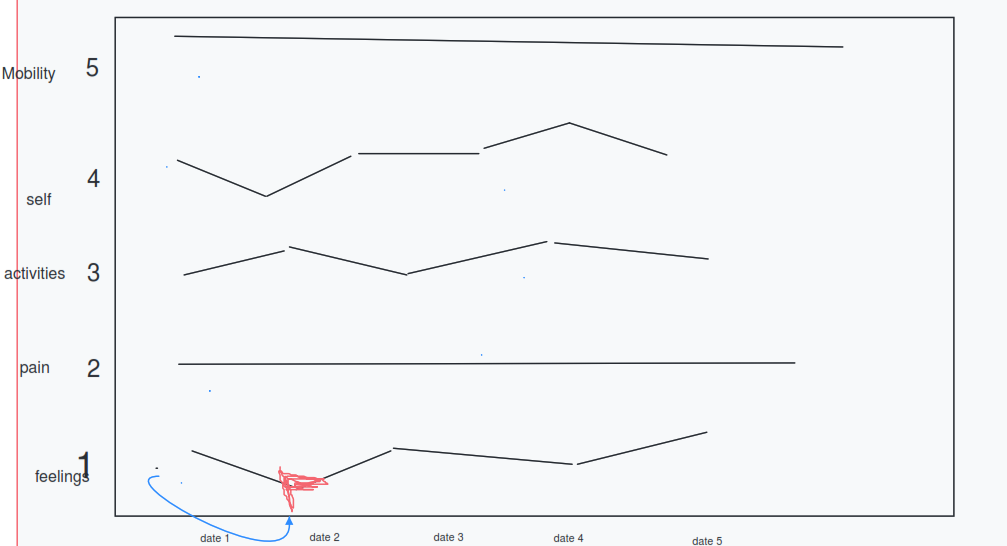


***Note:*** *This figure is an output from one of the workshops, it is an example display of EQ-5D-Y-5L responses over time drawn by a participant on Zoom Whiteboard.*

# Supplementary Figure 2. EQ-5D-Y-5L Introduction.

**General Health Tracking**

For an upcoming appointment with **[clinician name]** on [appointment date].

**Why am I being asked to answer these questions?**

We are asking you to answer these general health questions because you/ your child are taking part in the P-PROM ROCK research study and have an appointment coming up at The Royal Children’s Hospital.

We think it is important to understand how your/ your child’s overall health is going. This general health information can help the staff at the hospital to provide you/ your child with the best possible care.

# Supplementary Figure 3. Example of clinician display from a completed EQ-5D-Y-5L questionnaire (single time point table).

| **Questionnaires**  **EQ-5D-Y-5L, General Health Tracking Questionnaire** |  |
| --- | --- |
| **Question** | **19/02/24 12:54PM AEST**  Filled by child/patient |
| Who will fill out the questions today? | Child or patient |
| **Question** |  |
| MOBILITY | No problems walking around |
| LOOKING AFTER MYSELF | No problems washing or dressing self |
| DOING USUAL ACTIVITIES | Some problems doing usual activities |
| HAVING PAIN OR DISCOMFORT | No pain or physical discomfort |
| FEELING WORRIED SAD OR UNHAPPY | Quite worried, sad or unhappy |
| YOUR HEALTH TODAY (range 0 [0-the worst health imaginable] – 100 [100-best health imaginable]) | 86 |
| Thinking about the questions you have just answered, which of these would you like to talk to your doctor/nurse about at your upcoming appointment? | **DOING USUAL ACTIVITIES !** |

# Supplementary Figure 4. Example of clinician display from multiple completed EQ-5D-Y-5L questionnaires (over time, line graph).

***Note:*** *This is a mocked-up example of what the line graph over time looks like for clinicians in the electronic medical record system. Clinicians can click and select the EQ-5D-Y-5L items they would like to add to the line graph, in this example, all EQ-5D-Y-5L items have been selected. In this line graph, 1=worst health level (i.e., extreme problems/cannot do) and 5=best health level (i.e., no problems).*

# Supplementary Figure 5. Additional question used for children/caregivers to flag EQ-5D-Y-5L items to service providers.

Thinking about the questions you have just answered, which of these would you like to talk to your doctor/nurse about at your upcoming appointment? Tick all that apply.

- Mobility (walking around)
- Looking after self (washing or dressing)
- Doing Usual Activities (going to school, hobbies, sports, playing, doing things with family or friends)
- Having pain or discomfort
- Feeling worried, sad or unhappy
- General health scale from 0-100

# Supplementary Figure 6. Journey Map.

**
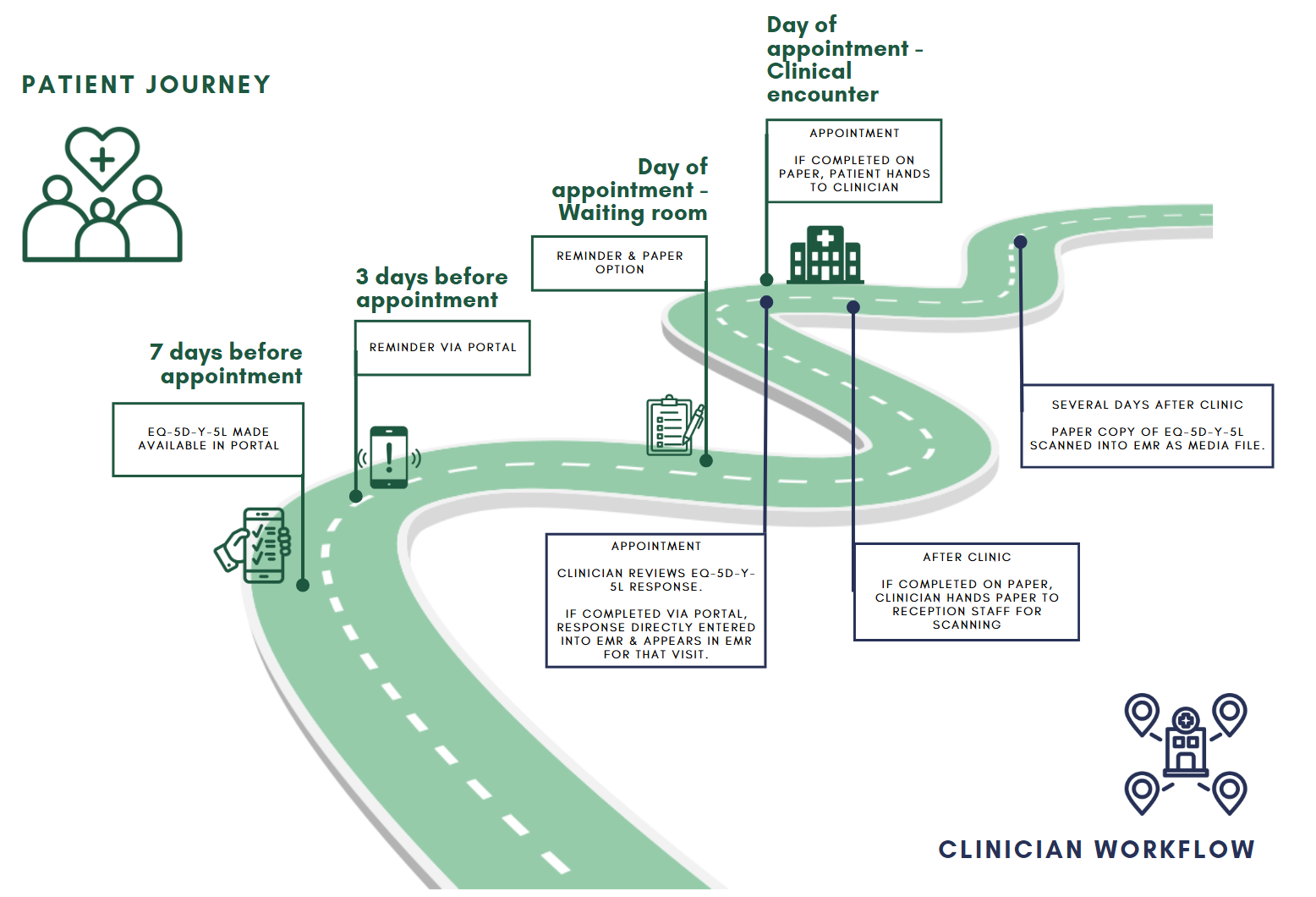
**

***Note:*** *Figure made in Canva (Canva.com).*

# Supplementary Figure 7. Example of Caregiver Resources.


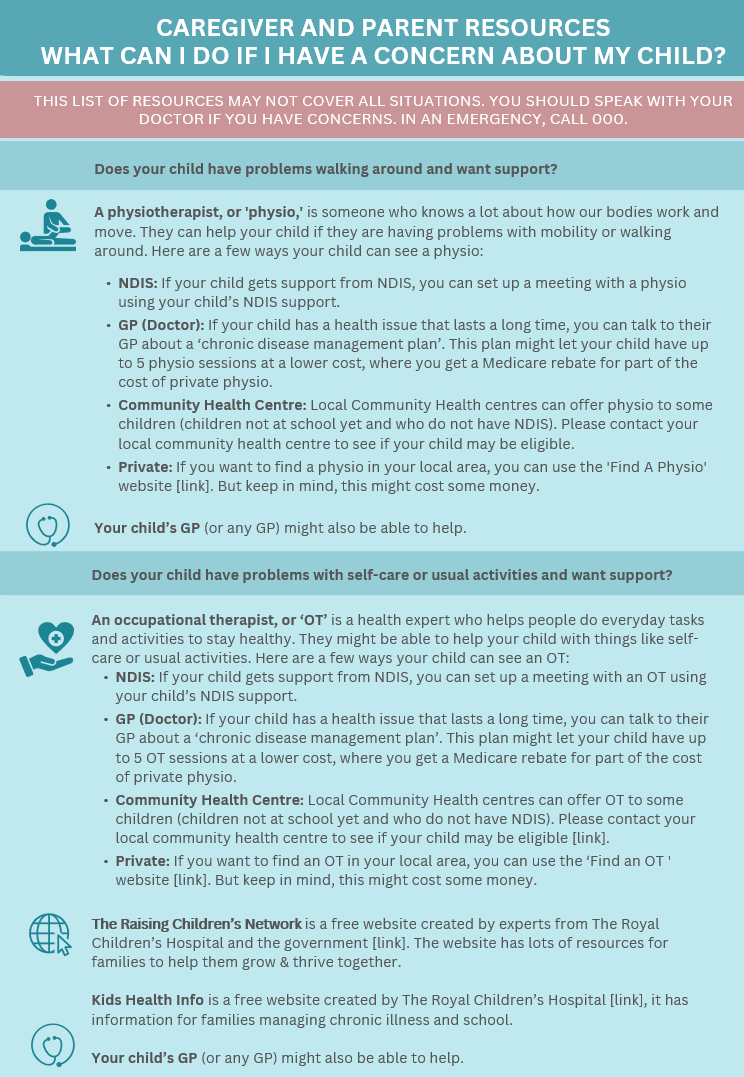


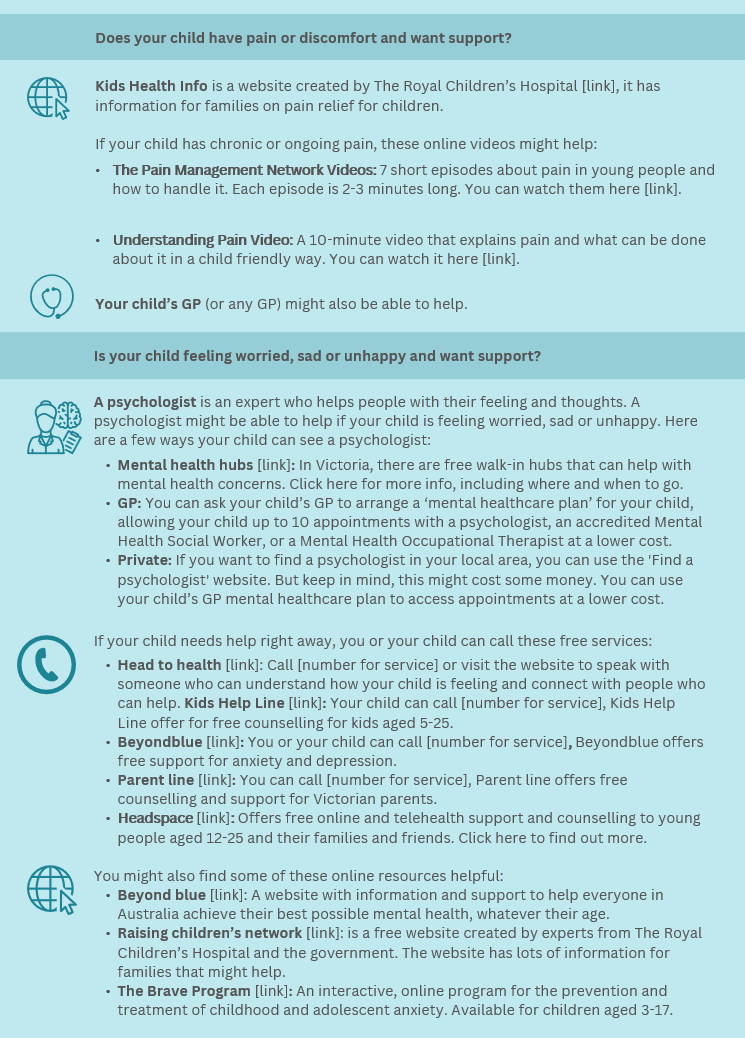


***Note:*** *Figure made in Canva (Canva.com). Website links, phone numbers and email addresses have been removed, this is denoted by square brackets.*

# Supplementary Figure 8. Clinician Decision Support Tool.


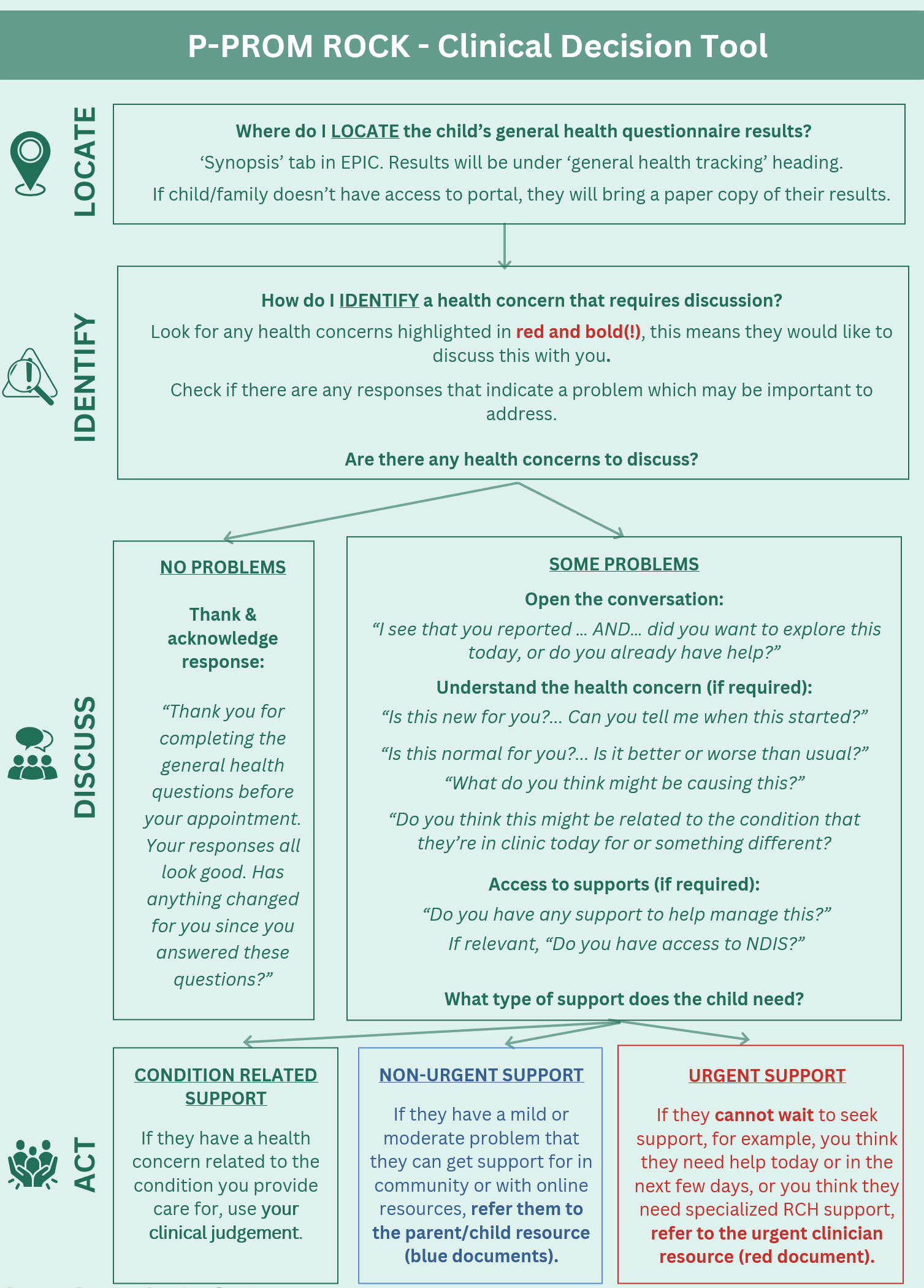


***Note:*** *Figure made in Canva (Canva.com).*

# Supplementary Figure 9. Clinician Resources for Urgent Concerns.

*
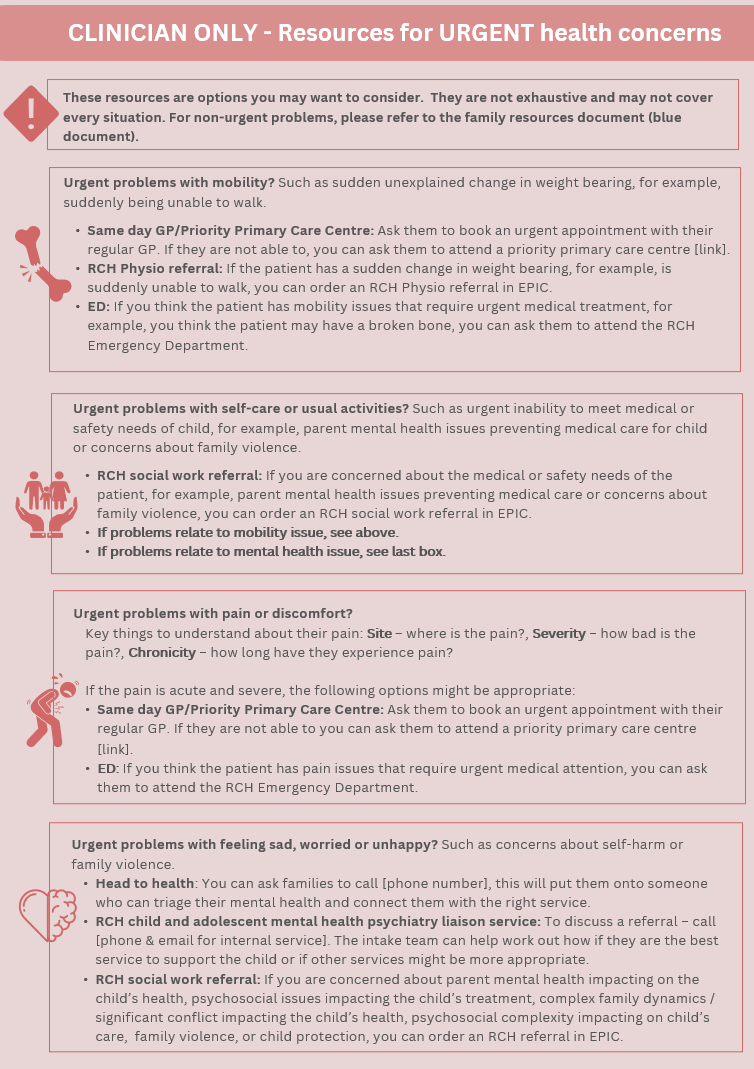
*

***Note:*** *Figure made in Canva (Canva.com). Website links, phone numbers and email addresses have been removed, this is denoted by square brackets.*
